# Diagnostic Value of Methylated *Septin9* for Colorectal Cancer Detection

**DOI:** 10.3389/fonc.2018.00247

**Published:** 2018-07-02

**Authors:** Li Xie, Xiyi Jiang, Qian Li, Zujun Sun, Wenqiang Quan, Yuping Duan, Dong Li, Tianhui Chen

**Affiliations:** ^1^Department of Clinical Laboratory, Shanghai First People’s Hospital, BaoShan Branch, Shanghai, China; ^2^Group of Molecular Epidemiology & Cancer Precision Prevention, Institute of Occupational Diseases, Zhejiang Academy of Medical Sciences, Hangzhou, China; ^3^Department of Clinical Laboratory, Shanghai Tongji Hospital, Tongji University School of Medicine, Shanghai, China; ^4^Department of Laboratory Medicine, Ninghai First People’s Hospital, Ningbo, China

**Keywords:** colorectal cancer, biomarker, *septin9*, methylation, early detection

## Abstract

**Background:**

Methylated *Septin9* (*mSEPT9*) has been suggested as a reliable biomarker in colorectal cancer (CRC) detection. We aimed to determine the diagnostic value of *mSEPT9* for CRC detection in Chinese patients. In addition, we compared the diagnostic efficacy of *mSEPT9* to traditional screening method [fecal occult blood test (FOBT)] and two biomarkers [carcinoembryonic antigen (CEA) and carbohydrate antigen-199 (Ca-199)].

**Methods:**

Overall 248 subjects including 123 patients with CRC and 125 controls were included. Plasma and fecal samples were collected for CEA, Ca-199, *mSEPT9*, and FOBT tests. Sensitivity and specificity were calculated to evaluate the diagnostic efficacy of each method; receiver operating characteristic (ROC) curve was plotted for the assessment of diagnostic accuracy, and comparisons among FOBT, *mSEPT9*, and the combination were assessed through area under the ROC curve (AUC).

**Results:**

*mSEPT9* achieved overall sensitivity and specificity of 61.8% [95% confidence interval (CI): 53.0–69.9%] and 89.6% (83.0–93.8%), respectively, with an AUC value of 0.757 (95% CI: 0.701–0.807), superior to FOBT [sensitivity: 61.4% (50.9–70.9%); specificity: 70.3% (59.1–79.5%); AUC: 0.658 (0.578–0.723)], CEA [sensitivity: 35.0% (27.1–43.7%); specificity: 62.6% (53.8–70.7%); AUC: 0.485 (0.411–0.559)], and Ca-199 [sensitivity: 17.9% (12.1–25.6%); specificity: 55.7% (48.9–64.1%); AUC: 0.353 (0.283–0.423)]. The combination of *mSEPT9* and FOBT further improved sensitivity and AUC value of 84.1% (75.1–90.3%) and 0.807 (0.752–0.863), respectively, while specificity was declined to 62.2% (50.8–72.4%).

**Conclusion:**

*mSEPT9* demonstrated best diagnostic ability in CRC detection compared with FOBT, CEA, and Ca-199. The combination of *mSEPT9* and FOBT further improved diagnostic sensitivity especially for early stage disease, which may provide a new approach for future CRC screening, though further investigations are warranted.

## Introduction

Colorectal cancer (CRC) is one of the most common gastrointestinal malignancies across the globe. According to GLOBOCAN 2012, CRC was the third most common cancer and the fourth leading cause of cancer-related death worldwide, accounting for approximately 1,360,000 new cases and 694,000 deaths per year (1). In China, the incidence and mortality rate of CRC both rank fifth among all malignant cancers (2); its incidence and mortality rate shall continue to increase along with the development of social economy and residents’ westernized lifestyle in China (2).

Screening has been shown to reduce CRC mortality (3, 4). Fecal occult blood test (FOBT) is widely used because of the low cost and non-invasiveness, whereas the sensitivity of FOBT alone for CRC screening is relatively low (commonly below 70%) due to many confounders (5, 6). Colonoscopy is currently considered as the gold standard for CRC detection (7, 8). However, high cost, invasive procedure and relatively high risk of complications preclude its wide implement especially in some undeveloped regions (9). Moreover, carcinoembryonic antigen (CEA) and carbohydrate antigen-199 (Ca-199) are used as blood-based tumor biomarkers but show unsatisfactory performance (10). Therefore, CEA and Ca-199 are not recommended for CRC screening while can be used for monitoring response to surgical or systemic therapy (11). Given that the ineffective or invasive of the traditional screening methods, blood-based biomarkers with high sensitivity, specificity, and compliance for CRC screening is urgently warranted for early detection of CRC.

It is well known that CRC occurs due to the genetic and epigenetic alterations of intestinal epidermal cells, experiencing three stages: molecular change, cellular change, and tissue change (12). Thus, the determination of specific molecular markers targeting the related changes may be a promising method for detecting early CRC (13). Recently, DNA methylation-based biomarkers have become a hot spot in cancer research. While DNA methylation is essential for the regulation of gene expression and the maintenance of cellular identity, epigenetic changes through altered DNA methylation play an important role in tumorigenesis (14). DNA methylation mainly occurs at C-phosphate-G (CpG) sites, in which unmethylated CpGs existing in clusters are called CpG islands. Aberrant methylation of CpG islands in promoter regions of genes has been linked to epigenetic transcriptional silencing of tumor suppressor genes, which appears to be crucial in the early stages of CRC development (15).

Several DNA methylation-based biomarkers for CRC have been reported in previous studies, of which, methylated *Septin9* (*mSEPT9*) is considered as a promising one for detecting CRC (16). *SEPT9*, located at chromosome 17q25.3, is a conservative skeletal protein gene with GTPas activity, involving in cytokinesis and cytoskeletal organization (17, 18). *SEPT9* is closely related to CRC carcinogenesis when the promoter region is hypermethylated and the transcription is compromised (19). The relationship between *mSEPT9* and CRC makes it possible to be used as an informative tumor biomarker. Specifically, *mSEPT9* DNA is released into the peripheral blood from necrotic and apoptotic cancer cells during CRC carcinogenesis; therefore, the risk of CRC can be determined by detecting the degree of DNA methylation of specific promoter region of *SEPT9* in the peripheral blood (20). Up to now, several studies have evaluated the diagnostic value of *mSEPT9* in CRC detection, yet the diagnostic accuracy differs significantly between each study, in which the sensitivity and specificity varied from 36.6 to 95.6% and 77 to 98.9%, respectively (20–24). Among them, few studies used Chinese CRC patients, with the sensitivity and specificity ranging from 69 to 88% and 87 to 98%, respectively (25–28). Therefore, it remains to be determined whether *mSEPT9* is a reliable biomarker for CRC detection in Chinese population.

In this study, we aimed to determine the diagnostic value of *mSEPT9* for blood-based CRC detection in a Chinese population. In addition, we compared the diagnostic efficacy of *mSEPT9* to traditional screening method (FOBT) and two blood-based tumor biomarkers (CEA and Ca-199), and in combinations among aforementioned biomarkers.

## Materials and Methods

### Study Subjects and Samples

All samples were collected from Affiliated Hospital of Tongji University and Baoshan branch of Shanghai First People’s Hospital. Subjects were recruited between October 1, 2016 and January 31, 2018. Only subjects who simultaneously performed CEA, Ca-199, and *mSEPT9* examinations were enrolled; and among them who received chemotherapy/surgical intervention were also excluded. Ultimately, overall 248 subjects were included in this study, including 123 CRC patients and 125 controls (diagnosed without CRC). Demographic and clinical–pathological information of subjects including sex, age, pathological type, tumor stage, and metastasis status were collected. CRC cases were confirmed by pathological diagnosis. Tumor stages were defined according to TNM staging system of 7th edition of the Cancer Staging Manual of American Joint Committee on Cancer (29).

This study was approved by the ethics committee of Affiliated Hospital of Tongji University and Baoshan branch of Shanghai First People’s Hospital. Collection of samples and clinical–pathological information from subjects were undertaken with informed consent.

### *SEPT9* Methylation Detection

#### DNA Preparation and Bisulfite Conversion From Plasma Specimens

For each sample, 3 mL blood was collected in an EDTA vacutainer tube (it was difficult to collect 10 mL blood, though 10 mL blood was initially requested for each subject). Each tube was immediately centrifuged at 1,350 × *g* for 10 min at room temperature. Plasma was transferred to a 2-mL tube without disturbing the pellet and stored at −20°C. The genomic DNA was extracted from 1 mL plasma using a Genomic DNA extraction Kit with magnetic beads from Tellgen Corporation, following the product protocol. Sample DNA was treated with bisulfite conversion reagents and purified by purification reagent, purchasing from Zymo Research (EZ DNA Methylation-Direct™ Kit) (30, 31). The unmethylated C bases in genomic DNA were modified to U bases by bisulfite, while the methylated C bases remained unchanged. Thus, the methylated and unmethylated C bases could be distinguished. For bisulfite conversion, we added 20 μL DNA (concentration between 10 and 50 ng/μL) to 130 μL CT Conversion Reagent (fresh prepared following the product protocol) in a 200-μL PCR tube and performed the conversion using a PCR program: pre-denaturation at 98°C for 8 min, CT Conversion at 64°C for 3.5 h, stored at 4°C up to 20 h. We purified bis-DNA using Zymo-Spin™ IC Column. Purified bis-DNA was eluted in 20-µL elution buffer (M-Elution Buffer) and used directly in methylation-specific real-time PCR (MSP) analysis.

#### Methylation-Specific Real-Time PCR

Methylated *Septin9* and beta-actin (ACTB) as internal control were performed in the same reaction. MSP was used to preferentially detect the methylated form of *SEPT9*. Following the bisulfite modification, MSP was performed with primers designed to amplify the methylated sequences in a part of *SEPT*9-v2 promoter. The DNA was analyzed by the Methylated Human *SEPT9* Gene Detection Kit (Tellgen Corporation) following the product protocol. The PCR mixtures contained 5 µL modified DNA, 15 µL PCR Mix including PCR reaction buffer, oligonucleotide primers, labeled probes and HotStart Taq DNA polymerase. The thermal cycling profile for PCR was set up as follows: pre-denaturation at 95°C for 10 min, 5 cycles of denaturation for 15 s at 95°C, annealing for 30 s at 60°C, and 40 cycles of denaturation for 15 s at 95°C, annealing for 32 s at 56°C. MSP was performed on the ABI7500 (Applied Biosystems).

#### PCR Data Analysis

PCR curves for the *mSEPT9* and ACTB were generated*. mSEPT9* was “detected” if the quantification cycle (“cycle threshold,” Ct) was less than 35 cycles. Plasma specimens were “not detected” if the *mSEPT9* Ct was not measurable or was ≥35 cycles and the ACTB Ct <35 cycles. If ACTB was not detected, sample DNA was treated with bisulfite conversion and MSP.

### Fecal Occult Blood Test

Colloidal gold immunochromatography with double antibody sandwich assay was used for the qualitative detection of fecal samples. A total of 8-mL fecal samples were detected using a WWT/FA160-Auto analyzer (Wowente Biotechnology Co. Ltd., Sichuan, China), with a 200 ng/mL hemoglobin cutoff test positivity.

### Carcinoembryonic Antigen

A total of 3–5 mL of venous blood was collected, and serum was isolated by centrifugation at 3,000 rpm for 15 min. Electronic chemiluminescence immunoassay (ECLIA) Kit (Roche Diagnostics GmbH) was used for CEA quantitative detection according to the manufacture’s instruction. A cutoff CEA value of 5.2 ng/mL was considered positive. Serum samples were detected immediately.

### Carbohydrate Antigen-199

A total of 3–5 mL of venous blood was collected, and serum was isolated by centrifugation at 3,000 rpm for 15 min. ECLIA Kit (Roche Diagnostics GmbH) was used for Ca-199 quantitative detection according to the manufacture’s instruction. A cutoff Ca-199 value of 39.0 U/mL was considered positive. Serum samples were detected immediately.

### Statistical Analysis

The differences of sex and age between case group and control group were analyzed using chi-square test and *t*-test, respectively. Detection results of *mSEPT9* were analyzed using positive rate and negative rate, and differences in detection rates among sex, age, and tumor location were analyzed using chi-square test or Fisher’s exact test. Sensitivity and specificity with 95% confidence intervals (CIs) were calculated to evaluate the diagnostic efficacy of each method (FOBT, CEA, Ca-199, *mSEPT9*, and the combination of FOBT and *mSEPT9*) in CRC detection. Meanwhile, diagnostic efficacies of methods of FOBT, *mSEPT9*, and the combinations in different tumor location, tumor stage, and metastasis status were evaluated and compared by sensitivity. Differences in sensitivity were analyzed using chi-square test or Fisher’s exact test. Furthermore, the receiver operating characteristic (ROC) curve was used to evaluate the diagnostic accuracy, and the comparisons among methods of FOBT, *mSEPT9*, and the combination were evaluated by the area under the ROC curve (AUC).

All statistical analyses were performed using the SAS statistical software, version 9.4 (SAS Institute Inc., Cary, NC, USA). Two-side *P*-value <0.05 was considered statistically significant.

## Results

### Demographic and Clinical Features of the Subjects

Table 1 presents basic characteristics on subjects. Overall 248 subjects were enrolled in the study, including 139 men (56.0%) and 109 women (44.0%). The median age was 66 years and the majority (73.4%) aged at least 60 years. Subjects in CRC case and control groups were 123 (74 men, 49 women, median age 66.07 years) and 125 (65 men, 60 women, median age 66.17 years), respectively. No significant differences in sex and age between the case group and control group were observed (*P* > 0.05). In the case group, 77 (62.5%) and 46 (37.5%) patients were diagnosed with colon cancer and rectum cancer, respectively. Within the colon cancer case group, tumor locations in sigmoid colon consisted of the largest proportion of 26.8% (33/123), compared with tumor locations in ileocecal junction (4.9%), ascending colon (6.5%), transverse colon (2.4%), splenic flexure colon (1.6%), descending colon (0.8%), and colon unspecified (19.5%). CRC patients in stage I, stage II, stage III, and stage IV were 5 (4.9%), 36 (35.0%), 58 (56.3%), and 4 (3.8%), respectively. In addition, CRC patients with regional lymph node metastasis, distant metastasis, and vascular and neural infiltration were 45, 6, and 25, respectively. In the control group, patients with bowel diseases consisted of the largest proportion of 40.8% (51/125), compared with the patients with malignancies in other systems (8.8%), other diseases (35.2%), and healthy control (15.2%).

**Table 1:** Demographic and clinical features of the subjects.

	Total (*n*, %)	CRC (*n*, %)	Control (*n*, %)
Sex^a^	248	123	125
Male	139 (56.0)	74 (60.2)	65 (52.0)
Female	109 (44.0)	49 (39.8)	60 (48.0)
Age (years)^b^			
<60	66 (26.6)	31 (25.2)	35 (28.0)
≥60	182 (73.4)	92 (74.8)	90 (72.0)
Median age (years)	66.12	66.07	66.17
Tumor location			
*Colon*	–	77 (62.5)	–
*Ileocecal junction*	–	6 (4.9)	–
*Ascending* colon	–	8 (6.5)	–
Transverse colon	–	3 (2.4)	–
Splenic flexure colon	–	2 (1.6)	–
Descending colon	–	1 (0.8)	–
Sigmoid colon	–	33 (26.8)	–
Colon, unspecified	–	24 (19.5)	–
Rectum	–	46 (37.5)	–
Non-CRC			
Bowel diseases		–	51 (40.8)
Malignancies in other systems		–	11 (8.8)
Other diseases		–	44 (35.2)
Healthy control		–	19 (15.2)
Tumor stage^c^			
Stage I		5 (4.9)	–
Stage II		36 (35.0)	–
Stage III		58 (56.3)	–
Stage IV		4 (3.8)	–
Regional lymph node metastasis^c^			
Yes		45 (43.7)	–
No		58 (56.3)	–
Distant metastasis^c^			
Yes		6 (5.8)	–
No		97 (94.2)	–
Vascular and neural infiltration^c^						
Yes		25 (24.3)	–
No		78 (75.7)	–

*CRC, colorectal cancer*.

*Bowel diseases including bowel polyp, bowel obstruction, and enteritis*.

*Malignancies in other systems including stomach cancer, lung cancer, liver cancer, pancreatic cancer, and breast cancer*.

*^a^P = 0.195*.

*^b^P = 0.956*.

*^c^Information was missing among 20 subjects (*n* = 103)*.

### Detection Results of *mSEPT9*

Overall 89 subjects were detected with *mSEPT9* positive, including 55 men (39.6%) and 34 women (31.2%); no sex difference was observed (Table 2). Older subjects (aged ≥60 years) had significantly higher positive rates of *mSEPT9*, compared with younger subjects (40.1 vs. 24.2%). CRC case group had significantly higher positive rates of *mSEPT9*, compared with the control group (61.8 vs. 10.4%). Within the CRC case group, patients with colon cancer had higher positive rates of *mSEPT9*, compared with rectum cancer (67.5 vs. 52.2%), though the difference did not reach statistical significance (*P* = 0.09). In the control group, patients with malignancies in other systems had significantly higher positive rates of *mSEPT9* (36.4%), compared with those with bowel diseases (9.8%) and with other diseases (9.1%). Notably, none of healthy control was detected with *mSEPT9* positive.

**Table 2:** Detection results of *mSEPT9* (*n* = 248).

	Positive (*n*, %)	Negative (*n*, %)	*P*-value
Sex	89	159	
Male	55 (39.6)	84 (60.4)	0.172
Female	34 (31.2)	75 (68.8)	
Age (years)			
<60	16 (24.2)	50 (75.8)	0.021*
≥60	73 (40.1)	109 (59.9)	
Tumor location^c^			
*Colon*	52 (67.5)	25 (32.5)	0.090^a^
Rectum	24 (52.2)	22 (47.8)	<*0.0001*^b^
Total	76 (61.8)	47 (38.2)	
Non-CRC^d^			
Bowel diseases	5 (9.8)	46 (90.2)	0.030*
Malignancies in other systems	4 (36.4)	7 (63.6)	
Other diseases	4 (9.1)	40 (90.9)	
Healthy control	–	19 (100.0)	
Total	13 (10.4)	112 (89.6)	

*mSEPT9, methylated Septin9; CRC, colorectal cancer*.

*P < 0.05 indicates statistically significant.

*^a^Comparation between patients of colon cancer and rectum cancer*.

*^b^Comparation between subjects in CRC group and non-CRC group*.

*^c^n = 123*.

*^d^n = 125*.

### Diagnostic Efficacy of Each Method

As is shown in Table 3, *mSEPT9* showed the highest diagnostic ability with a sensitivity of 61.8% (95% CI: 53.0–69.9%), compared with FOBT [61.4% (50.9–70.9%)], CEA [35.0% (27.1–43.7%)], and Ca-199 [17.9% (12.1–25.6%)] (*P* < 0.05). Meanwhile, *mSEPT9* also had the highest specificity [89.6% (83.0–93.8%)], compared with FOBT [70.3% (59.1–79.5%)], CEA [62.6% (53.8–70.7%)], and Ca-199 [55.7% (48.9–64.1%)] (*P* < 0.05). Notably, the combination of *mSEPT9* with FOBT further improved sensitivity, reaching 84.1% (75.1–90.3%), though the specificity declined [62.2% (50.8–72.4%)].

**Table 3:** Diagnostic efficiency of each method in CRC detection.

	Sensitivity (%)		Specificity (%)		AUC	
	Value	95% CI	Value	95% CI	Value	95% CI
FOBT^a^	61.4	50.9–70.9	70.3	59.1–79.5	0.658	0.578–0.723
CEA	35.0	27.1–43.7	62.6	53.8–70.7	0.485	0.411–0.559
Ca-199	17.9	12.1–25.6	55.7	48.9–64.1	0.353	0.283–0.423
*mSEPT9*	61.8	53.0–69.9	89.6	83.0–93.8	0.757	0.701–0.807
FOBT + *mSEPT9*^a^	84.1	75.1–90.3	62.2	50.8–72.4	0.807	0.740–0.875

*FOBT, fecal occult blood test; CEA, carcinoembryonic antigen; Ca-199, carbohydrate antigen-199; AUC, area under the ROC curve; 95% CI, 95% confidence interval; mSEPT9, methylated Septin9; CRC, colorectal cancer*.

*^a^FOBT information was missing among 86 subjects*.

Table 4 presents further stratifications by tumor location. *mSEPT9* had higher sensitivity for colon cancer compared with rectum cancer (67.5 vs. 52.2%; *P* > 0.05). *mSEPT9* had higher sensitivity for cancer at right colon compared with cancer at left colon (71.4 vs. 66.7%; *P* > 0.05); for right colon, *mSEPT9* had higher sensitivity for ascending colon cancer, compared with ileocecal junction cancer (75.0 vs. 66.7%; *P* > 0.05). For colon cancer, *mSEPT9* achieved higher sensitivity, compared with FOBT for overall (67.5 vs. 57.8%; *P* > 0.05), right colon (71.4 vs. 63.6%; *P* > 0.05), and left colon (66.7 vs. 59.3%; *P* > 0.05), also similar performance for tumor locations of ileocecal junction, ascending colon, sigmoid colon, and unspecified colon (*P* > 0.05) (Table 4). However, for rectum cancer, *mSEPT9* had lower sensitivity, compared with FOBT (55.2 vs. 67.7%; *P* > 0.05). Notably, when combined by *mSEPT9* with FOBT, the sensitivity further improved, reaching 86.0% for colon and 80.7% for rectum. Actually, the sensitivity improved for all stratifications except for transverse colon (remaining the same 66.7%). For instance, the sensitivity reached 90.9% for right colon (100% for ileocecal junction), and 92.6% for left colon (96.0% for sigmoid colon).

**Table 4:** Detection sensitivity of fecal occult blood test (FOBT), *mSEPT9*, and the combination for the stratification by tumor location (%).

	FOBT^a^	*mSEPT9*	FOBT + *mSEPT9*^a^
Colon (*n* = 77)	57.8	67.5	86.0
*Right colon (n* = *14)*	63.6	71.4	90.9
Ileocecal junction (*n* = 6)	50.0	66.7	100.0
Ascending colon (*n* = 8)	71.4	75.0	85.7
*Left colon (n* = *36)*	59.3	66.7	92.6
Splenic flexure and descending colon (*n* = 3)	50.0	0	50.0
Sigmoid colon (*n* = 33)	60.0	72.7	96.0
*Transverse colon (n* = *3)*	66.7	66.7	66.7
*Colon, unspecified (n* = *24)*	50.0	66.7	75.0
Rectum (*n* = 46)	67.7	52.2	80.7

*^a^FOBT information was missing among 20 patients in group of colon cancer, including 2 in ileocecal junction, 1 in ascending colon, 1 in descending colon, 8 in sigmoid colon, and 8 in unspecified colon, and FOBT information was missing among 15 patients in group of rectum cancer*.

Table 5 presents the stratifications by tumor stage and tumor metastasis. *mSEPT9* had slightly lower sensitivity compared with FOBT for overall stage (59.2 vs. 63.6%; *P* > 0.05), and for stages I–III, except for stage IV, but the number was very small (4 cases) for stage IV. *mSEPT9* had higher sensitivity compared with FOBT for patients with regional lymph node metastasis (60.0 vs. 54.3%; *P* > 0.05), for patients with distant metastasis (83.3 vs. 33.3%; *P* > 0.05), and for patients with vascular and neural infiltration (56 vs. 52.9%; *P* > 0.05). Interestingly, *mSEPT9* had higher sensitivity for patients with distant metastasis, compared with those without (83.3 vs. 57.7%; *P* > 0.05), though the number was small for patients with distant metastasis (*n* = 6). When combined by *mSEPT9* with FOBT, the sensitivity significantly improved, reaching 85.7% for overall, 100.0% for stage I, 82.6% for stage II, 88.9% for stage III, and 50.0% for stage IV. Besides, when combined by *mSEPT9* with FOBT, the sensitivity improved for all subgroups, reaching 85.7% for patients with regional lymph node metastasis, 83.3% for patients with distant metastasis, and 82.4% for patients with vascular and neural infiltration.

**Table 5:** Detection sensitivity of fecal occult blood test (FOBT), *mSEPT9*, and the combination for the stratification by tumor stage and metastasis (%).

	FOBT^a^	*mSEPT9*	FOBT + *mSEPT9*^a^
Stage (*n* = 103)	63.6	59.2	85.7
Stage I (*n* = 5)	80.0	60.0	100.0
Stage II (*n* = 36)	56.5	52.8	82.6
Stage III (*n* = 58)	68.9	63.8	88.9
Stage IV (*n* = 4)	25.0	50.0	50.0

Regional lymph node metastasis (*n* = 103)			
Yes (*n* = 45)	54.3	60.0	85.7
No (*n* = 58)	71.4	58.6	85.7

Distant metastasis (*n* = 103)			
Yes (*n* = 6)	33.3	83.3	83.3
No (*n* = 97)	66.2	57.7	85.9

Vascular and neural infiltration (*n* = 103)			
Yes (*n* = 25)	52.9	56.0	82.4
No (*n* = 78)	66.7	60.3	86.7

*^a^FOBT information was missing among 26 patients, including 13 in group of stage II and 13 in stage III; FOBT information was missing among 10 and 8 patients in groups of regional lymph node metastasis (Yes) and vascular and neural infiltration (Yes), respectively, and FOBT information was missing among 16, 26, and 18 patients in groups of regional lymph node metastasis (No), distant metastasis (No), and vascular and neural infiltration (No), respectively*.

### ROC Curve Analysis of FOBT, *mSEPT9*, and the Combination

**Figure 1:**
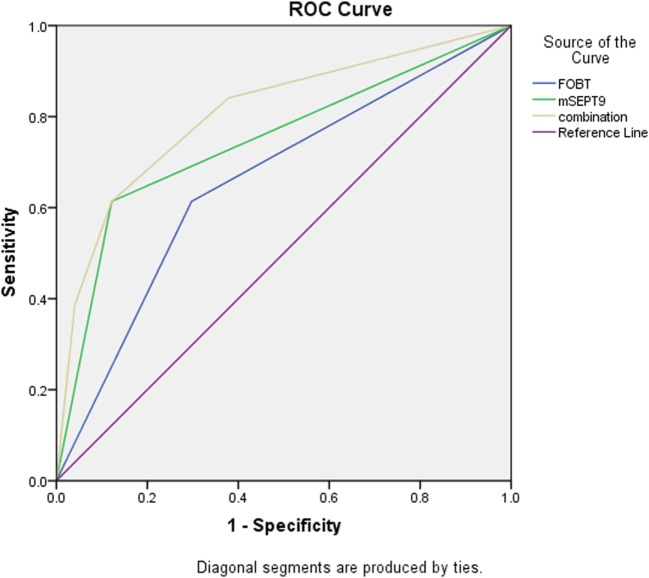
Receiver operating characteristic (ROC) curve of fecal occult blood test (FOBT), methylated *Septin9* (*mSEPT9*), and the combination.

As is shown in Table 3 and Figure 1, *mSEPT9* achieved the highest AUC value of 0.757 (95% CI: 0.701–0.807), comparing to FOBT [0.658 (0.578–0.723)], CEA [0.485 (0.411–0.559)], and Ca-199 [0.353 (0.283–0.423)]. Besides, the combination of *mSEPT9* with FOBT further improved the AUC value, reaching 0.807 (0.752–0.863).

## Discussion

In this study, we evaluated the diagnostic value of *mSEPT9* for blood-based CRC detection in Chinese patients, compared with traditional screening method (FOBT) and two blood-based tumor biomarkers (CEA and Ca-199). We found *mSEPT9* achieved overall sensitivity of 61.8% (53.0–69.9%) and specificity of 89.6% (83.0–93.8%), with an AUC value of 0.757 (0.701–0.807), superior to FOBT [sensitivity: 61.4% (50.9–70.9%); specificity: 70.3% (59.1–79.5%); AUC: 0.658 (0.578–0.723)], CEA [sensitivity: 35.0% (27.1–43.7%); specificity: 62.6% (53.8–70.7%); AUC: 0.485 (0.411–0.559)], and Ca-199 [sensitivity: 17.9% (12.1–25.6%); specificity: 55.7% (48.9–64.1%); AUC: 0.353 (0.283–0.423)]. The combination of *mSEPT9* with FOBT further improved sensitivity [84.1% (75.1–90.3%)] and AUC value [0.807 (0.752–0.863)], though the specificity declined [62.2% (50.8–72.4%)].

Colorectal cancer is a common gastrointestinal malignancy with poor prognosis and high mortality rate, for which screening and early detection are crucial especially for high-risk population. Investigations on blood-based biomarkers for early detection of CRC are highly warranted because stool-based tests are not convenient. Our results indicated a poor performance of CEA and Ca-199 for detecting CRC, suggesting that they shall not be recommended for CRC screening, in line with previous studies (10, 11). Apart from the traditional biomarkers, growing evidence has shown a significant association between *mSEPT9* and CRC pathogenesis, indicating the potential role of *mSEPT9* in CRC detection (32). *mSEPT9* is a promising biomarker in CRC detection, with the advantage of convenience and non-invasiveness. Nevertheless, diagnostic sensitivity of *mSEPT9* in CRC detection varied among published literatures. For European and American population, the sensitivity ranged from 58 to 95.6% (20, 22–24, 33–35); the sensitivity varied from 69 to 88% in Asia population, which was higher compared with our results (25–27). Yet, Lee et al. (21) found a very low sensitivity of *mSEPT9* (36.6%) among Korean population. Interestingly, we found *mSEPT9* can be detected both in men and women for each age group and non-CRC diseases, with a higher positive rate among men and older subjects (aged ≥60 years). Therefore, differences in sensitivity may be partly attributed to the sample heterogeneity caused by demographic characteristics (sex, race, and age), lifestyles (smoking and alcohol consumption), comorbidities, or other environmental exposure factors (36).

Methylated *Septin9* can be detected in tumor locations of colon and rectum. *mSEPT9* had higher sensitivity for colon cancer compared with rectum cancer (67.5 vs. 52.2%), in line with the investigation conducted by Li et al. (37). Besides, a higher sensitivity was found for cancer at right colon compared with cancer at left colon (71.4 vs. 66.7%), which was similar to the findings from Li et al. (37). The reason may be partly attributed to different carcinogenesis for colon and rectum cancers.

We found *mSEPT9* can be detected among all stages and the sensitivity reached highest (63.8%) for stage III disease, consisted with the study by Jin et al. (38) showing a higher sensitivity for patients in stage III and stage IV and with the study by Ørntoft et al. (39) showing a higher sensitivity for patients with stage II–IV diseases. In addition, we found higher sensitivity for patients with advanced stage, such as metastasis (regional lymph node metastasis/distant metastasis). Our findings demonstrate that CRC patients with advanced stage are more easily detected by *mSEPT9* compared with those with early stage.

We found the combination of *mSEPT9* with FOBT further improved the overall sensitivity [84.1% (75.1–90.3%)] and AUC value [0.807 (0.752–0.863)], in line with the study by Johnson et al. (40) showing the combination of *mSEPT9* and FOBT further improved sensitivity (88.7%) and with the study by Wu et al. (41) reporting of 94.4% sensitivity for the combination. For the stratification by tumor location, we found the combination further improved the sensitivity both for colon and rectum. Notably, we found 100.0% sensitivity for tumor location of ileocecal junction and for CRC patients in stage I. However, the number was small (*n* = 6 for patients with ileocecal junction cancer and *n* = 5 for patients in stage I), further investigations with large number are highly warranted. Furthermore, the combination also improved the sensitivity for patients with advanced stage (with metastasis and infiltration). Taken together, our findings suggest that *mSEPT9* could be a complementary tool (in addition to FOBT) for CRC detection.

This study has several strengths and limitations. First, CRC cases were confirmed by pathological diagnosis, which guarantee the diagnostic accuracy. Second, diagnostic efficacy of each method in different groups of tumor location, tumor stage, and metastasis were evaluated and compared, which could provide guidance for clinical application in the future. Major limitation concerns sample size. Small number for the stratifications might lead to an over-evaluated diagnostic value especially for some subgroups of tumor location. For instance, patients with rectum caner only accounted for a small proportion. Thus, further investigations with large number are warranted. Second, small volume (3 mL) of plasma samples were used for only one time detection, which was smaller compared with previous studies (both for the blood volume and detection number). Therefore, the results especially the sensitivity might be influenced. Third, while 35% (86/248) subjects had missing value for fecal test results, the missing value was randomly distributed among case group but not among all subjects. Thus, the results might be biased. Furthermore, this study focuses on the diagnostic value of *mSEPT9* in CRC detection, yet the association between *mSEPT9* and prognosis of CRC patients is unknown, thus further study is needed.

In conclusion, we found *mSEPT9* achieved overall sensitivity of 61.8% (53.0–69.9%), specificity of 89.6% (83.0–93.8%), and AUC value of 0.757 (0.701–0.807), superior to FOBT, CEA, and Ca-199. The combination of *mSEPT9* with FOBT further improved sensitivity, though the specificity decreased. Therefore, further investigations on the diagnostic performance of the fecal test by different cutoff values are highly warranted. Along with the advantage of convenience and non-invasiveness, *mSEPT9* is a promising biomarker in CRC detection. It could be expected that a further colonoscopy targeting the high-risk population (*mSEPT9* positive) could improve the confirmation ability of CRC diagnosis, which avoids the repeated screening and reduces the invasive procedures. Combined use of *mSEPT9* with traditional method of FOBT could improve the diagnostic sensitivity especially among CRC patients in early stage, which may provide a new approach for future CRC screening, while further investigations with large sample size are highly warranted.

## Ethics Statement

This study was approved by the ethics committee of Affiliated Hospital of Tongji University and Baoshan branch of Shanghai First People’s Hospital. Collection of samples and clinical-pathological information from subjects were undertaken with informed consent.

## Author Contributions

LX and DL were responsible for the study concept and design. TC and DL obtained funding. LX and DL acquired data. LX and XJ analyzed and interpreted data. LX, XJ, and TC drafted the manuscript, and all authors revised it for important intellectual content. DL and TC are the guarantors of this work.

## Conflict of Interest Statement

The authors declare that the research was conducted in the absence of any commercial or financial relationships that could be construed as a potential conflict of interest.
